# Towards measuring effective coverage: critical bottlenecks in quality- and user-adjusted coverage for major depressive disorder in São Paulo metropolitan area, Brazil

**DOI:** 10.1186/s13033-023-00583-w

**Published:** 2023-06-16

**Authors:** Mariane Henriques Franca, Chrianna Bharat, Ercole Novello, Irving Hwang, Maria Elena Medina-Mora, Corina Benjet, Laura Helena Andrade, Daniel V. Vigo, Maria Carmen Viana

**Affiliations:** 1 Post-Graduate Program in Public Health, Federal University of Espirito Santo, Vitória, ES Brazil; 2 National Drug & Alcohol Research Centre, Faculty of Medicine, University of New South Wales, Sydney, NSW 2052 Australia; 3 SYNLAB Computing Center, Barcelona Area, Spain; 4 Department of Health Care Policy, Harvard Medical School, Boston, MA USA; 5 National Institute of Psychiatry Ramón de la Fuente, Mexico City, Mexico; 6 Department of Epidemiologic and Psychosocial Research, National Institute of Psychiatry Ramón de la Fuente Muñiz, Mexico City, Mexico; 7 Nucleo de Epidemiologia Psiquiatrica, Departamento e Instituto de Psiquiatria, Hospital das Clinicas da Faculdade de Medicina da Universidade de Sao Paulo-LIM 23, Sao Paulo, Brazil; 8 Department of Psychiatry, University of British Columbia, Vancouver, BC Canada; 9 Department of Global Health and Social Medicine, Harvard Medical School, Boston, MA USA; 10 Department of Social Medicine and Post-Graduate Program in Public Health, Federal University of Espirito Santo, Vitoria, ES Brazil

**Keywords:** Depressive disorder, Treatment coverage, Effective treatment, Bottlenecks

## Abstract

**Background:**

Major depressive disorder (MDD) contributes to a significant proportion of disease burden, disability, economic losses, and impact on need of treatment and health care in Brazil, but systematic information about its treatment coverage is scarce. This paper aims to estimate the gap in treatment coverage for MDD and identify key bottlenecks in obtaining adequate treatment among adult residents in the São Paulo Metropolitan area, Brazil.

**Methods:**

A representative face-to-face household survey was conducted among 2942 respondents aged 18+ years to assess 12-month MDD, characteristics of 12-month treatment received, and bottlenecks to deliver care through the World Mental Health Composite International Diagnostic Interview.

**Results:**

Among those with MDD (n = 491), 164 (33.3% [SE, 1.9]) were seen in health services, with an overall 66.7% treatment gap, and only 25.2% [SE, 4.2] received effective treatment coverage, which represents 8.5% of those in need, with a 91.5% gap in adequate care (66.4% due to lack of utilization and 25.1% due to inadequate quality and adherence). Critical service bottlenecks identified were: use of psychotropic medication (12.2 percentage points drop), use of antidepressants (6.5), adequate medication control (6.8), receiving psychotherapy (19.8).

**Conclusions:**

This is the first study demonstrating the huge treatment gaps for MDD in Brazil, considering not only overall coverage, but also identifying specific quality- and user-adjusted bottlenecks in delivering pharmacological and psychotherapeutic care. These results call for urgent combined actions focused in reducing effective treatment gaps within services utilization, as well as in reducing gaps in availability and accessibility of services, and acceptability of care for those in need.

## Background

The Global Burden of Disease (GBD) has thrown light on the huge impact of mental disorders in society as they are the leading cause of disability, with an associated massive economic and societal burden [1, 2]. Major depressive disorder (MDD), owing to its high lifetime prevalence and its associated significant disability, presents the highest societal burden. In 2013, MDD was the second largest cause of disease burden across the world [1], and the number of incident cases of MDD increased by 49.86% from 1990 to 2017 worldwide [3]. In Brazil, MDD is the fifth major cause of disability-adjusted life years (DALY), contributing to a significant proportion of disease burden, disability, economic losses, and impact in treatment and health care [4–6]. Although effective treatments for MDD have been widely demonstrated, societal strategies to mitigate these burdens can play a crucial role in the implementation of cost-effective interventions, reducing clinical severity and comorbidity, and preventing chronicity and disability [7–10].

Monitoring treatment gaps, barriers and bottlenecks for those in need of the population in need is essential for tracking progress towards universal health coverage, an important aim for of the UN’s Sustainable Development Goals (SDG’s) [11]. As coverage indicators provide no indication about the quality of interventions and their potential for ameliorating symptoms, however, there is increasing evidence that national coverage indicators may overstate the health benefits of the health care systems because of poor quality of services [12, 13]. For this reason, there is a need to shift from research focused on contact coverage to effective coverage, accounting for the quality of services and their impact on individual’ health.

The definition and measurement of effective coverage varies between studies and there is a need for standard terminology and methods for coverage measurement. We use the effective coverage cascade framework developed by Vigo et al. [14], adapted for the World Health Organization World Mental Health (WHO-WHM) Surveys [14–18], which was based on Tanahashi’s framework [15]. Building upon previous WHO-WMH studies that analyzed minimally adequate treatment for MDD [19] Vigo et al.’s [14] framework intended to understand the “effective coverage” indicator through adjustments for quality of care (i.e., inputs and process) and user adherence (i.e., to physician indications) [17, 20].

The effective coverage cascade framework distinguishes actual and potential coverage. Actual coverage is related to service output, and is a composed of contact coverage (% of people in need that get any service care) and effective coverage (% that get quality care). Potential coverage refers to all service capacity when services are available, accessible, and acceptable to users in need. Effective coverage refers to the percentage of people who do get good care and obtain health benefits from it, with favorable outcomes expected; this measure is a function of both quality of care delivered and users’ adherence [14, 15]. Following this view, Vigo et al. [14] analyzed the main bottlenecks in contact coverage among individuals with DSM-IV major depressive disorder in the 12 months (12-mo) prior to interview (12-mo MDD) using surveys conducted across 15 countries by the World Health Organization WMH Survey Initiative, including the São Paulo Megacity Survey from Brazil. The prevalence of 12-mo MDD was 4.8%, and among those with MDD 41.8% received any mental health services, and only 23.2% were considered effective treatment. The gap in effective treatment was 90% due to lack of utilization (58%) and inadequate quality or adherence (32%). Critical bottlenecks were related to underutilization of psychotherapy, underutilization of psychopharmacology, inadequate physician monitoring, and inadequate drug-type. Severe cases were more likely than mild-moderate cases to receive either adequate pharmacotherapy or psychotherapy, but less likely to receive an adequate combination of both, i.e., an effective coverage [14].

The Brazilian Mental Health System is composed of The Unified Health System (SUS), that ensures universal access coverage within the country, and a larger private health care sector, which provides mainly private care, but may be contracted to deliver public care under government control [21, 22]. Twenty-six percent of Brazilians have private health insurance and although the coverage is concentrated in the urban areas of the South-eastern part of the country, its coverage is growing across the nation [21]. Socioeconomic inequalities boost barriers to extend health care systems in Brazil, which are also unequally distributed across the country, including the mental health care system. The Mental Health Care System in Brazil is essentially community-based provided by the Community Social Psychiatric Centres, labelled *Centro de Atenção Psicosocial* (CAPS), which essentially substituted psychiatric beds after the Brazilian Psychiatric Reform [22]. CAPS are responsible for severe mental disorders treatment as well as to articulate the liaison with primary health care to provide treatment for common mental disorders [22].

Accessibility is related to the population’s socioeconomic status in Brazil, which combined with the growing health care load of the elderly population, increases the burden upon mental health care services, contributing to the huge treatment gap of mental disorders [22–25]. Socioeconomic inequalities may shape the differences in mental health treatment practices, impacting mental health outcomes in individuals with mental disorders, especially with depression and anxiety disorders [25–29], as poorer developments are related to differences in readiness of treatment access and quality of care [25, 27, 28].

Using the Vigo et al.’s [14] framework, Brazilian data were analyzed separately, aiming to produce specific estimates for the São Paulo Metropolitan Area, in Brazil: (a) treatment contact coverage among individuals with 12-mo MDD (pharmacotherapy, psychotherapy, and combination of both), and their association with severity; (b) partially adequate and adequate use of antidepressants among individuals with 12-mo MDD receiving psychotropic medication, and their association with severity; (3) partially adequate and adequate use of psychotherapy among individuals with 12-mo MDD receiving any psychotherapy, and their association with severity. Based on these results, we developed and analyzed the effective coverage cascade for MDD in the Sao Paulo Metropolitan area, Brazil.

## Methods

### Sample

The São Paulo Megacity is a multi-stage cross-sectional population-based epidemiological study designed to assess psychiatric morbidity in a representative sample of adult household residents aged 18 or older living in the São Paulo metropolitan area (SPMA), with a global response rate of 81.3% [30]. Data were collected between May/2005 and April/2007 by trained lay interviewers, using the paper and pencil version of the World Mental Health Survey Composite International Diagnostic Interview (CIDI 3.0), a fully structured diagnostic interview that is composed of clinical and non-clinical sections arranged in Part I and Part II [31]. Core disorders (anxiety, mood, impulse-control, and substance use disorders) and sociodemographic risk factors were assessed in all respondents (Part I sample). WMH-CIDI non-core clinical modules as well as non-clinical sections were administered in a subsample composed by all core disorder cases and a 25% random sample of non-cases (Part II sample). A total of 5037 Part I and 2942 Part II individuals were interviewed and we focus our analyses on the 491 Part II individuals with 12-mo MDD.

### Measures

#### 12-month major depressive disorder

Major depressive episode among respondents who did not have a lifetime history of bipolar spectrum disorder [32] occurring in the 12 months prior to the interview were assessed through the CIDI 3.0 clinical sections, based on the Diagnostic and Statistical Manual of Mental Disorders, 4th Edition (DSM-IV) diagnostic criteria (12-mo MDD) [33].

Severity of 12-mo MDD*:* MDD severity were classified into three categories: (1) *severe* if their depression resulted in severe role impairment (7–10 points) according to the Sheehan Disability Scale (SDS) [34]; (2) *moderate* if they reported moderate role impairment in the SDS (4–6 points), and (3) *mild* if they reported no or moderate role impairment (3 or less).

#### Service use

Respondents were asked how many visits in the past 12 months they made to a psychiatrist, medical doctor, psychologist, social worker, counselor, mental health professional, non-mental health professional, for any mental health or substance-use problems. They were asked also if they stopped seeing these providers, and if they completed the full recommended course of treatment.

Health treatment providers*:* Respondents were classified into two categories, according to the health services used in the past 12 months: (1) *specialist mental health* (SMH: psychiatrist, psychologist, other mental health professional in any setting, social worker, or counselor in a mental health specialized setting); and (2) *general medical services* (GM: primary care doctor, other medical doctor, any other healthcare professional seen in a GM setting). For the purposes of these analyses, we did not include or consider help sought from spiritual advisors or healers.

*Contact coverage* was defined as having had any contact with a SMH specialist or GM provider for a mental health condition in the past 12-months.

#### 12-month contact coverage and treatment provided

If respondents saw a medical provider in the past 12 months, they were asked about type of treatment received, i.e., pharmacotherapy, psychotherapy, or both.

### Pharmacotherapy

For each psychotropic medication used in the past 12 months, specific class of drug, dose, and duration were recorded [14]. If respondents reported more than 3 medications, they were further asked about three random medications, out of the maximum of 10 reported, and medications were categorized as anti-depressants, mood-stabilizers, anti-psychotics, and other (any other psychotropic medication). Respondents were then classified into two categories: (1) receiving *any psychotropic medication*; and (2) *receiving any antidepressant*. Respondents were also asked how many days out of 30 they either forgot to take the medication or took less than prescribed, in a typical month over the past 12 months. *Patient adherence* with medication use was defined as not having missed or taking less than was prescribed for 3 days or more in a typical month [35–37]. *Medication control* was classified as *Adequate* if respondents used any psychotropic medications and had at least four visits with any physician or psychiatrist [14, 38].

According to these variables several combinations were constructed, as follows:

Adequate pharmacotherapy: Classified as such if respondents were (1) taking antidepressants with adequate medication control by any physician and adequate patient adherence; or (2) taking any non-antidepressant psychotropic medication with adequate medication control by a psychiatrist and adequate patient adherence.

Pharmacotherapy for antidepressants: considered as (1) *Partially adequate pharmacotherapy for any antidepressants,* defined as having 2 of the 3 conditions: appropriate medication (antidepressants); and/or adequate medication control for the anti-depressant treatment; and/or patient adherence for the antidepressant use. (2) *Adequate pharmacotherapy for any antidepressants*: defined as having all 3 of the above-described conditions.

### Psychotherapy

*Any psychotherapy* was considered if respondents had two or more visits to a psychiatrist for, on average, 30 or more minutes; or two or more visits to another SMH provider [14]. The adequate number of sessions was defined as at least 8 sessions over the past 12 months [14, 38]. Psychotherapy adherence depended on whether the respondent prematurely ended treatment [14].

*Adequate psychotherapy* was considered if respondents had at least 8 sessions from a SMH provider; or if they are still in treatment after 2 visits. Visits to psychiatrists needed to last 30 min or more to be considered as psychotherapy (and not merely medication control) [14]. Partially Adequate psychotherapy: Considered as above, but with a minimum of 5 sessions or 2 or more visits and still in treatment.

### Effective coverage

Effective coverage, i.e. an adequate treatment combination, was adjusted for MDD severity, and defined as (1) respondents with mild and moderate MDD having received adequate pharmacotherapy and/or adequate psychotherapy; or (2) respondents with severe MDD having received a combination of both adequate pharmacotherapy and adequate psychotherapy [14, 38–44].

### Contact coverage cascade framework

According to the variables described above, we developed the contact coverage cascade framework including adjustments for quality-of-care (inputs and processes) and user adherence (physician prescription, drug dosage, and early drop out) [14]. To identify critical bottlenecks, we analyzed the relative size of each gap in the context of the whole effective coverage cascade and focused our analysis on gaps that represent a drop of 10 percentage points or more in overall coverage for MDD cases.

### Analysis

Descriptive statistics and measurements of associations were calculated applying survey sampling procedures that consider the complex sampling design, using weights to adjust for sampling probabilities and for the age-sex structure of the target-population; the Part II sample was further weighted for the under sampling of Part I respondents without core disorders [30].

The proportion estimates and standard errors of people with 12-mo MDD who received treatment were calculated using the Taylor series linearization method [45] implemented in SAS (version 8.2, SAS Institute, Cary, N.C.). Effects of weighting and clustering on significance tests for these subgroup differences were adjusted for using the Taylor series linearization method [45].

The statistical significance of differences in conditional treatment prevalence estimates by disorder severity was evaluated with logistic regression models, with dummy control variables for age, sex, and marital status. Statistical significance was evaluated using 0.05 level, two-sided tests.

## Results

The description of the sample, according to sociodemographic and clinical severity among respondents with 12-mo MDD is showed in Table 1. A total of 491 respondents fulfilled DSM-IV criteria for a 12-mo MDD episode (with a prevalence estimate of 9.4%, SE 0.6—published elsewhere [46]). About three fourths of the respondents with 12-mo MDD were female, over 70% were younger than 45 years of age, over half were married, less than 20% had a high education, less than 40% had a private health insurance, 61.9% were working, and about 46% had a severe MDD clinical presentation over the past 12 months.

**Table 1:** Sample characteristics regarding sociodemographic and clinical severity among respondents with 12-mo MDD; Sao Paulo Metropolitan Area, Brazil (Part II sample N = 2942)

	12-mo DSM-IV MDD N = 491		
	n	%	SE
Sex			
Male	122	24.9	2.91
Female	369	75.1	2.91
Age group			
18–29	125	33.2	2.96
30–44	198	40.6	3.06
45–59	133	20.8	1.70
60+	35	5.5	1.29
Marital status			
Separated, divorced or widowed	120	22.5	2.08
Never married	83	23.5	2.69
Married or cohabiting	288	54.0	2.63
Income quartiles			
Low	135	24.3	2.26
Low-average	133	25.7	2.12
Average-high	112	25.4	3.00
High	111	24.7	2.85
Level of education			
Low	114	17.8	1.85
Low-average	131	25.9	2.76
Average-high	168	37.9	2.66
High	78	18.4	2.03
Insurance			
Any private insurance	188	38.0	2.19
Only public health (SUS)	303	62.0	2.19
Employment status			
Working	268	61.9	2.50
MDD clinical severity			
Severe	226	45.8	2.25
Moderate	180	36.8	2.18
Mild	85	17.4	1.71

MDD, major depressive disorder; SE, standard error

Unweighted numbers (n) and weighted percentages (%) are shown

### Twelve-month health system contact coverage for 12-mo MDD

Contact coverage over the past 12 months for respondents diagnosed with a 12-mo MDD according to clinical severity and type of treatment received is presented in Table 2. From those with MDD (N = 491), a total of 164 (33.3% [SE, 1.9]) had 12-month contact coverage. Considering clinical severity, individuals with severe MDD were more likely to receive any psychotropic medication than those with mild/moderate presentations (37.2% [SE, 4.4] vs 17.8% [SE, 2.5]; F = 14.0, p = < 0.001). They were also more likely to receive antidepressants (21.8% [SE, 3.8] vs 12.7% [SE, 2.3]; F = 5.4, p = 0.029); adequate medication control (20.2% [SE, 3.0] vs 10.0% SE, 1.9]; F = 10.3, p = 0.004); psychotherapy (19.9% [SE, 2.9] vs 8.8% SE, 1.6]; F = 22.6, p = < 0.001); and adequate psychotherapy (14.9% [SE, 2.8] vs 8.2% SE, 1.6]; F = 10.0, p = 0.004) (Table 2).

**Table 2:** Contact coverage among all diagnosed with 12-mo MDD (N = 491), according to MDD clinical severity; Sao Paulo Metropolitan Area, Brazil

	12-mo DSM-IV MDD						F (p-value)
	Severe (n = 224)		Mild/moderate (n = 265)		Any severity (n = 489)^a^		
	n	% (SE)	n	% (SE)	n	% (SE)	
Contact coverage	90	42.1 (3.8)	74	26.6 (3.1)	164	33.3 (1.9)	7.00 (0.014)*
Any psychotropic medication	79	37.2 (4.4)	47	17.8 (2.5)	126^#^	26.1 (2.3)	13.99 (< .001)*
Any antidepressants	52	21.8 (3.8)	32	12.7 (2.3)	84^#^	16.6 (2.2)	5.35 (0.029)*
Adequate medication control	43	20.2 (3.0)	21	10.0 (1.9)	64	14.3 (1.9)	10.26 (0.004)*
Adequate pharmacotherapy	25	11.1 (2.6)	12	6.1 (1.9)	37	8.3 (1.7)	3.08 (0.09)
Any psychotherapy	39	19.9 (2.9)	22	8.8 (1.6)	61	13.5 (1.9)	22.57 (< 0.001)*
Adequate psychotherapy	30	14.9 (2.4)	19	8.2 (1.6)	49	11.1 (1.7)	10.04 (0.004)*
Effective coverage	13	7.3 (1.8)	22	9.2 (1.8)	35	8.4 (1.5)	0.92 (0.35)

MDD, major depressive disorder; SE, standard error

Unweighted numbers (n) and weighted percentages (%) are shown

*Significant at the 0.05 level, two-sided test

^a^Two 12-mo MDD cases were exclude due to lack of information regarding service use

^#^Eleven respondents were in use of medication without a 12-month prescription

### Treatment for 12-mo MDD among those with any 12-month contact coverage

Table 3 shows treatment received for 12-mo MDD, according to clinical severity, among those with any 12-month contact coverage, analyzing the use of pharmacotherapy, psychotherapy, and the adequate combination of both, i.e., effective coverage. Among those receiving treatment: (a) 63.6% [SE, 3.1] received any psychotropic medication, but only 24.8% [SE, 4.6] received adequate pharmacotherapy; (b) 40.7% [SE, 5.0] received psychotherapy, but only one-third received adequate psychotherapy; and (c) 25.2% [SE, 4.2] received a severity-adjusted adequate combination of pharmacotherapy and/or psychotherapy (Table 2). Considering MDD clinical severity, only 17.3% [SE, 4.3] of severely affected individuals and 34.5% [SE, 7.1] of those with mild to moderate MDD received an adequate combination of psychotherapy and pharmacotherapy, and this difference was statistically significant (Table 3).

**Table 3:** Treatment for DSM-IV 12-mo MDD, according to clinical severity, among those with any 12-month contact coverage (N = 164); São Paulo Metropolitan Area, Brazil

Any 12-month contact coverage	12-Mo DSM-IV MDD						F (p-value)
	Severe (n = 90)		Mild/Moderate (n = 74)		Any severity (n = 164)		
	n	% (SE)	n	% (SE)	n	% (SE)	
Any psychotropic medication	66	71.0 (5.4)	39	54.8 (6.7)	105	63.6 (3.1)	2.30 (0.14)
Any antidepressants	47	47.2 (6.7)	26	40.1 (7.7)	73	43.9 (5.2)	0.51 (0.48)
Adequate medication control	43	48.0 (7.0)	21	37.4 (7.4)	64	43.1 (4.9)	0.95 (0.34)
Adequate pharmacotherapy	25	26.3 (5.7)	12	22.9 (7.4)	37	24.8 (4.6)	0.13 (0.72)
Any psychotherapy	39	47.3 (6.5)	22	33.0 (6.2)	61	40.7 (5.0)	2.98 (0.10)
Adequate psychotherapy	30	35.5 (5.8)	19	30.9 (6.3)	49	33.4 (4.5)	0.32 (0.58)
Effective coverage	13	17.3 (4.3)	22	34.5 (7.1)	35	25.2 (4.2)	5.36 (0.029)*

MDD, major depressive disorder; SE, standard error

Unweighted numbers (n) and weighted percentages (%) are shown

*Significant at the 0.05 level, two-sided test

### Partially and adequate use of antidepressants and psychotherapy for 12-mo MDD among individuals receiving psychotropic medication and psychotherapy

A more detailed assessment of the quality of treatment delivered is shown in Table 4, among individuals with 12-mo MDD receiving antidepressant medication and/or psychotherapy, by clinical severity.

**Table 4:** Treatment for 12-mo MDD among individuals receiving antidepressants and/or psychotherapy over the past 12 months, by clinical severity; São Paulo Metropolitan Area, Brazil

	12-mo DSM-IV MDD and contact coverage						F (p-value)
	Severe (n = 90)		Mild/moderate (n = 74)		Any severity (n = 164)		
	n	% (SE)	n	% (SE)	n	% (SE)	
Among those with 12-mo MDD, receiving antidepressant medication^a^	47	47.2 (6.7)	26	40.1 (7.7)	73	43.9 (5.2)	0.51 (0.48)
At least partially adequate pharmacotherapy^1^	37	81.0 (7.6)	23	95.5 (3.1)	60	87.1 (5.1)	4.13 (0.05)*
Adequate pharmacotherapy^2^	21	48.3 (12.0)	11	56.7 (12.8)	32	51.8 (9.0)	0.23 (0.63)
Among those with 12-mo MDD, receiving any psychotherapy^a^	39	47.3 (6.5)	22	33.0 (6.2)	61	40.7 (5.0)	2.98 (0.10)
At least partially adequate psychotherapy^3^	32	79.3 (8.5)	19	93.6 (4.6)	51	84.6 (5.7)	2.16 (0.15)
Adequate psychotherapy^4^	30	75.0 (9.2)	19	93.6 (4.6)	49	81.9 (6.2)	3.17 (0.09)

MDD, major depressive disorder; SE, standard error

Unweighted numbers (n) and weighted percentages (%) are shown

*Significant at the 0.05 level, two-sided test

^a^Data from Table 3 in row

^1^Partially adequate pharmacotherapy for antidepressants, defined as having 2 of the 3 (appropriate medication—antidepressant), appropriate medication control (4 or more visits to an MD) for antidepressants, and adherence to treatment (defined as < = 3 days of not taking medication in a typical month) for anti-depressants

^2^Adequate pharmacotherapy for antidepressants, defined as having all 3: appropriate medication (antidepressant), and appropriate medication control (4 or more visits to an MD) for the antidepressant, and adherence to treatment (defined as < = 3 days of not taking medication in a typical month) for antidepressants

^3^Partially adequate psychotherapy: seeing (a psychiatrist for 5 or more visits to a Psychiatrist AND on average 30 or more minutes) OR (5 or more visits to any other MH provider) OR (2 or more visits to psychiatrist AND on average 30 or more minutes AND still in treatment) or (2 or more visits to any other MH provider AND still in treatment)

^4^Adequate psychotherapy: seeing (a psychiatrist for 8 or more visits to a Psychiatrist AND on average 30 or more minutes) OR (8 or more visits to any other MH provider) OR (2 or more visits to a psychiatrist AND on average 30 or more minutes AND still in treatment) or (2 or more visits to any other MH provider AND still in treatment)

A total of 69.0% [SE, 7.1] of the individuals receiving any psychotropic medication were prescribed antidepressant medication (Tables 3 and 4). Among them, 87.1% [SE, 5.1] received at least partially adequate pharmacological treatment, and, of those, 51.8% [SE, 9.0] received adequate pharmacotherapy (Table 4). Considering severity, individuals with severe MDD were significantly less likely to receive at least partially adequate pharmacotherapy than individuals with mild or moderate MDD (81.0% [SE, 7.6] vs 95.5% [SE, 3.1], F = 4.13; p = 0.05). From those receiving any psychotherapy, 84.6% [SE, 5.7] and 81.9% [SE, 6.2] received at least partially adequate and adequate psychotherapy, respectively, with no differences for clinical severity (Table 4).

### Main bottlenecks in coverage

**Fig. 1:**
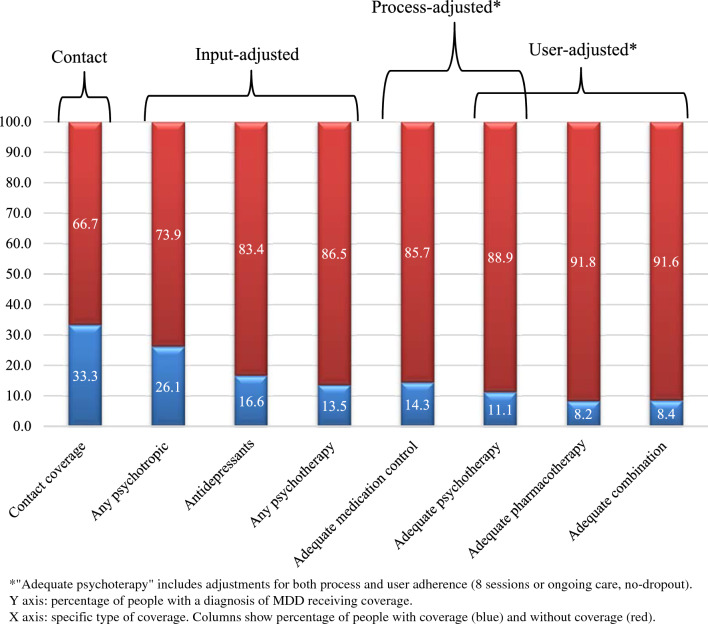
Overall contact coverage, quality-adjusted (input and process) coverage, and used-adjusted coverage for 12-mo MDD. Sao Paulo Metropolitan Area, Brazil. MDD, major depressive disorder

Overall contact coverage for MDD, quality-adjusted (input and process, i.e., types of treatment provided and adequate follow-up by provider), and user-adjusted (i.e., adherence to treatment) coverage is depicted in Fig. 1. Only 33.3% of people in need received any treatment (contact coverage), and only 8.4% of MDD cases received effective coverage (i.e., quality- and user-adjusted coverage). This represents a 91.6% gap for effective treatment coverage, which can be decomposed into 66.7% due to lack of contact and 24.9% (91.6–66.7%) due to inadequate quality and adherence (Fig. 1).

#### Pharmacotherapy bottlenecks

Among those who had made treatment contact, only 21.1% (N = 105 Table 3, Fig. 1; see footnote in Table 2) received any medication from any health provider. Among those with MDD and contact coverage over the past 12 months, the main bottlenecks related to use of medication were:

1. Use of any psychotropic medication by 63.6% (N = 105), i.e., a bottleneck of 12.2 percentage points (33.3–21.1%, Fig. 1) relative to those who had made contact with health services over the past 12 months (N = 164), which represents a relative drop of 36.6%;
2. Only 69.0% (N = 73) of those receiving psychotropic medication received antidepressants**,** representing a bottleneck drop of 6.5 (21.1–14.6%—Fig. 1) percentage points in the input-adjusted coverage (i.e., adequate medication prescribed), which represents a relative drop of 30.8% in the psychopharmacological treatment offered, when adequacy of medication (i.e., prescription of antidepressants) is taken into account;
3. The use of any psychotropic medication (63.6%) and being adequately monitored by a physician (67.8%), representing a bottleneck drop of 6.8 percentage points in process-adjusted (i.e., adequate medication control) coverage (21.1–14.3%, Fig. 1); this represents a 32.2% relative drop in the initial psychopharmacological treatment offered, when quality of treatment is taken into account; and
4. Only 39.0% (24.8 × 100/63.6, Table 3) received adequate pharmacotherapy, a drop of 12.9 percentage points, which represents a 61.1% relative drop when process, input and user-adjusted coverage is taken into consideration over initial treatment with psychotropic medication (Fig. 1).

#### Psychotherapy bottlenecks

Of the 33.3% of people with MDD and contact coverage only 40.7% received any psychotherapy (Table 3), a drop of 19.8 percentage points in input-adjusted coverage (Fig. 1: 33.3–13.5%). In terms of process and adherence, 82.0% (33.4 × 100/40.7) of the psychotherapy provided was considered adequate, a drop of only 2.4 percentage points (Fig. 1), representing a 17.8% relative drop when quality of treatment and adherence are considered.

## Discussion

Quite a small proportion of individuals with 12-mo MDD received effective treatment coverage in Metropolitan São Paulo, the largest metropolitan area in South America. Our findings confirm that Brazil has a huge unmet need of MDD care, with critical bottlenecks in effective treatment with both, underutilization of pharmacotherapy and psychotherapy. Indeed, less than 1 in 10 people with MDD received quality- and user-adjusted coverage (8.4%), defined by a combination of adequate pharmacological and psychotherapy treatment received, with user adherence. This gap was determined by specific bottlenecks: a drop of 12.2, 18.7 and 19.8 percentage points in receiving any medication, any antidepressants and any psychotherapy, respectively, among 12-month help-seekers; and a further drop of 12.9 percentage points for adequate antidepressant treatment among those receiving medication, and 2.4 percentage points for adequate psychotherapy, for those receiving it. Our overall coverage framework highlights the bottlenecks and, therefore, the potential directions for improving quality of care and effective coverage, in accordance with the SDG of achieving universal health coverage, including for mental health and wellbeing [11, 14].

Contact coverage for MDD reached only 33.3% of the population in need. This may be due to lack of availability and accessibility of services, as well as individual perception of acceptability or stigma that influences help-seeking behavior. Furthermore, even when services are reached, inadequate care was often provided.

Previous research in Brazil, demonstrated that less than 25% of individuals with mental disorders actually obtained access to services [47]. Apart from structural barriers, the main psychological reasons reported for not seeking treatment were the low perception that treatment is necessary and the willingness to resolve the problem on their own, without professional help [47]. Previous studies have indicated that less than 20% of people with MDD recover on their own, highlighting the importance of the availability of mental health services and quality of care delivered [48].

Results from 15 countries participating in the WHO-WMH surveys initiative have shown that only 23.6% received effective treatment, among the 52% of individuals with 12-mo MDD that had contact coverage in high-income countries, while among the 26.5% of individuals with 12-month MDD and contact coverage in low- or middle-income countries, including Brazil, only 21.7% received effective care. Critical bottlenecks were related to underutilization of psychotherapy and of psychopharmacology, inadequate physician monitoring, and inadequate drug-type used [14].

In Brazil, the largest challenges in improving MDD treatment effectiveness among individuals who had contact coverage would come from improving physician monitoring of medication and increasing referral and utilization of psychotherapy: nearly 64% of individuals with MDD are being prescribed psychotropics, and only 67.8% of them are being adequately monitored by a physician, and only 40.7% are receiving any psychotherapy. Psychological therapies, such as cognitive behavior therapy, and antidepressants, occasionally enhanced with antipsychotics, have proven beneficial for treating depression [49]. Digital interventions to manage MDD cases have proven to decrease depressive symptoms, improve self-reported quality of life, treatment adherence, and recovery [50] and may increase scalability of services especially in the context of additional barriers to treatment during the COVID-19 pandemic.

However, the main bottleneck and largest treatment gap, i.e., those in need who do not reach services, remain untouched by current public policies, and cases cannot be identified and managed outside the health services framework. Therefore, apart from improving the quality of treatment coverage, there is a pressing need for increasing service facilities, and therefore, increasing treatment availability and accessibility, as well as expanding population awareness of depressive symptoms and acceptability of seeking help.

Considering the financial burden of depression [5, 6] as well as the cost-effectiveness of pharmacotherapy and psychotherapy in the world [51], especially in Brazil, this huge unmet need in the availability and quality of treatment coverage for MDD in Brazil can be explained, in part, by structural deficiencies, such as inadequate and insufficient infrastructure, as well as a severe shortage of qualified mental health professionals, capable of delivering effective care [22], and highlights the challenges imposed by mental disorders upon the Brazilian health system. Most of the changes implemented in the public mental health system do not concern the treatment of MDD, as primary care professionals are not systematically trained to manage such conditions. Furthermore, primary care or general health care professionals may not feel as comfortable to treat mental disorders as physical illnesses or may discredit the efficacy of psychotropic medication or psychotherapy [52]. Mental health professionals are not usually included in primary care teams, and additionally, there is a lack of integration between primary care and mental health care settings which is a challenge for improving the quality of medication control by physicians and increasing referrals for psychotherapy. As recently as 2005, there were only 6003 psychiatrists working in the public Unified Health System in Brazil (3.26 per 100,000 population), and most were concentrated in larger cities. The number of psychologists was higher (10.2 per 100,000 population), but little is known about what types of interventions were delivered and which psychiatric disorders were managed [22]. It is known that Brazilian social policies have endured shortage of funding as well as political indifference in recent years, and mental health policy makers have neglected the importance of community-based mental health care. All these factors combined may further affect the supply of services and have a deleterious impact on the already huge needs to improve quality of physician mental health care and to scale up psychotherapeutic services, which would be necessary to increase the availability and accessibility of effective coverage.

Several environmental and socio-economic characteristics, as well as health system arrangements and clinical guidelines adopted, may explain these huge treatment gaps for depression observed in this study: geographic and demographic characteristics; insurance coverage, social security, and other forms of public benefits; the availability and distribution of the mental health workforce and pharmaceuticals; as well as culturally determined health-related attitudes and behaviors. Further knowledge on how these variables impact the bottlenecks identified herein can provide additional insights for policy makers on the appropriate societal response and funding allocation that should be placed to reduce unmet needs.

## Limitations

Our results should be considered within the limitations of our study design. Data on service utilization relied on self-reports that may be subject to recall bias, although we focused our assessments on the 12-month period before the interview to minimize this risk. Social desirability bias could also affect some measures, as respondents may be reluctant to acknowledge non-adherence. We could not adopt more stringent methods to assess respondents’ adherence, such as drug concentration in blood samples or pill counting, as such measures are unfeasible for population-level cross-sectional household surveys. The diversity of therapeutic practices may not be fully captured by standardized indicators that include, for example, a uniform threshold for the number or duration of visits. It may be possible that pharmacotherapy and/or psychotherapy can be effectively delivered in less than the proposed number or duration of visits considered as adequate in this study, as our classification was based on a review of the April 2018 National Institute for Health and Care Excellence Guidelines [53] the 2016 Canadian Network for Mood and Anxiety Treatments guidelines [41, 42], the 2010 American Psychiatric Association Practice Guideline for the Treatment of Patients with Major Depressive Disorder [43], and the 2016 WHO Mental health gap guide Intervention Guide [44].

Further, it is likely that some respondents qualified for comorbid mental disorders, and it cannot be ruled out that the comorbid disorder, rather than the depressive episode, was the exclusive focus of the treatment received. In practice though, clinicians treat people, rather than specific diagnoses and CIDI-diagnosed MDD can be expected to be a key component of most comorbid clinical presentations that include depression, and it is unlikely to be overshadowed and excluded from the focus of care. Hence, this study works under the assumption that the type of quality- and adherence-adjusted care we analyzed would, in people that fulfill MDD DSM-IV diagnostic criteria, address MDD as a meaningful component of comorbid clinical presentations. Still, it should be noted that our results do not address mental or physical comorbidities, and how it may affect service needs. Finally, because a small fraction of people with MDD may be prescribed non-antidepressant psychotropics, due to side effects, failed trials, or other off-label drug use [14], we also considered the use of non-antidepressant psychotropics, with adequate control by a psychiatrist and adequate patient adherence, as adequate interventions; this analytic procedure may have increased the effective coverage levels, and, therefore, the bottlenecks could be even higher.

## Conclusions

This is the first study demonstrating the huge treatment gaps for MDD in Brazil, considering not only overall coverage, but also identifying specific quality- and user-adjusted bottlenecks on delivering pharmacological and psychotherapeutic care. These results call for urgent combined actions focused in reducing effective treatment gaps within services utilization, as well as in reducing gaps in availability and accessibility of services, and acceptability of care for those in need.

## Data Availability

Public access to the diagnostic instrument, including diagnostic algorithms, should be requested via: http://www.hcp.med.harvard.edu/wmh. Nevertheless, there are limitations on the availability of raw data due to ethical restrictions related to sensitive information and to the signed agreement with the WHO World Mental Health Survey Initiative to limit comparative analyses to those carried out within the consortium. Requestors wishing to access a de-identified minimal dataset necessary for only monitoring purposes of our published analyses can apply to Dr Maria Carmen Viana: mcviana6@gmail.com.

## Abbreviations

MDD: Major depressive disorder
GBD: Global Burden of Disease
DALY: Disability-adjusted life years
SDG’s: UN’s Sustainable Development Goals
WHO-WMH: World Health Organization World Mental Health
DSM-IV: Diagnostic and Statistical Manual of Mental Disorders, fourth edition
12-mo: 12 Months
SUS: Unified Health System
CAPS: Centro de Atenção Psicosocial
SPMA: Sao Paulo metropolitan area
CIDI 3.0: Composite International Diagnostic Interview
WMH: World Mental Health
SDS: Sheehan disability scale
SMH: Specialist mental health services
GM: General medical services
OR: Odds ratio
